# Is the sublay self-gripping mesh effective for incisional ventral hernia repair? Our experience and a systematic review of the literature

**DOI:** 10.1007/s13304-020-00762-1

**Published:** 2020-05-08

**Authors:** Elena Schembari, Maria Sofia, Rosario Lombardo, Valentina Randazzo, Ornella Coco, Edoardo Mattone, Gaetano La Greca, Domenico Russello, Saverio Latteri

**Affiliations:** 1 General Surgery, Cannizzaro Hospital, University of Catania, Via Messina 829, 95126 Catania, Italy; 2 Department of Medical Surgical Sciences and Advanced Technologies “Ingrassia”, University of Catania, General Surgery Unit, Cannizzaro Hospital, Via Messina 829, 95126 Catania, Italy; 3 General Surgery Unit, Cannizzaro Hospital, Via Messina 829, 95126 Catania, Italy

**Keywords:** Self-gripping mesh, Incisional hernia, Sublay

## Abstract

Sublay mesh repair seems to be the most effective method for treating incisional hernias (IHs). The aim of this study was to report our experience with retromuscular repair and self-gripping mesh for the treatment of midline IHs. In addition, we provided a systematic review of the literature regarding the use of this novel combination. All patients undergoing elective IH repair from June 2016 to November 2018 were included. The self-gripping mesh was placed in the sublay position. Demographic data, defect sizes, postoperative complications and follow-up durations were collected. A systematic review of the available literature was conducted in January 2020 using main databases. A total of 37 patients (20/17M/F) were included in this study, and the mean age and body mass index (BMI) were 58 years and 27 kg/m^2^, respectively. Minor complications occurred in six patients. Long-term follow-up demonstrated recurrence in three patients. Regarding the review, five publications were considered relevant. The highest complication rate was 28.6%, and the recurrence rate varied from 0 to 5.1%. This is the first review of the literature regarding sublay IH repair using a self-gripping mesh. The low rates of postoperative complications and recurrence in our experience and those reported by most of the reviewed articles demonstrate that this is a safe and effective method for repairing IHs.

## Introduction

Incisional hernia (IH) is a common late complication of laparotomy, with an estimated occurrence rate of 12.8% after approximately 2 years [1]. Mesh repair seems to be more effective than suture repair in the treatment of IHs because the former leads to a lower recurrence rate [2]. Currently, there is no consensus regarding the technique or the type of mesh that should be used [3, 4]. However, sublay mesh placement seems to have the lowest recurrence rate [5, 6]. This is probably due to the position of the mesh between the posterior rectus sheath and the anterior myofascial complex, which provides the tissues that are necessary for mesh integration [5, 7]. Regarding the type of mesh, there is a novel polyester mesh with resorbable polylactic acid microgrips that adheres to the area of the repair better than other types of meshes [8]. Several studies have suggested the safety and efficacy of self-gripping mesh inguinal hernia repair [9, 10]. However, only a few articles have described the outcomes of the sublay placement of this mesh type in IH repair. The aim of this study was to analyse data derived from our experience and the literature to demonstrate the safety and efficacy of the sublay self-gripping mesh technique as a sutureless and tension-free treatment option for the open repair of midline IHs. In addition, we conducted a systematic review of the literature to support our experience.

## Materials and methods

A retrospective, single-centre study was performed from June 2016 to November 2018. All patients with an IH were included. The exclusion criteria were patients under 18 years and pregnant females as well as patients with signs of infection and with strangulated hernias. The diagnosis of a hernia was based on the clinical examination performed at the outpatient clinic. In cases of doubtful diagnosis, the diagnosis was confirmed by CT. All patients provided informed consent for hernia repair before surgery. The patients’ age, sex, body mass index (BMI), smoking status, comorbidities (ASA score) (Table 1), number of previous surgical operations, defect location and size (Table 2), mesh size, postoperative complications and follow-up duration data were collected.

**Table 1:** Demographic data for the 37 patients considered in this study

Variables	Data
Mean age (years)	58 ± 13 years (median 57 years; range 13–85 years)
Sex	17 (46%) females: 20 (54%) males
Mean BMI (kg/m^2^)	27 ± 12.5 kg/m^2^ (median 26.3 kg/m^2^; range 18–35 kg/m^2^)
Smoking	9 (24%)
ASA score	
ASA I	10 (27%)
ASA II	18 (48.6%)
ASA III	9 (24.4%)

*BMI *body mass index,* ASA *American Society of Anesthesiologists

**Table 2:** Hernia defect characteristics according to the European Hernia Society (EHS) classification

	Number of patients
Hernia defect characteristics	
M2M3M4M5	2 (5.4%)
M1M2M3	2 (5.4%)
M2M3M4	13 (35.2%)
M3M4M5	1 (2.7%)
M2M3	3 (8.1%)
M3M4	6 (16.2%)
M4M5	1 (2.7%)
M2	2 (5.4%)
M3	4 (10.8%)
M4	1 (2.7%)
M5	2 (5.4%)
Width	
W1 (< 4 cm)	4 (10.8%)
W2 (≥ 4–10 cm)	19 (51.4%)
W3 (≥ 10 cm)	14 (37.8%)

### Operative details

All operations were performed under general anaesthesia after antibiotic administration of 875/125 mg amoxicillin/clavulanic acid. Another dose of the same antibiotic was administered 12 h after the operation. The peritoneal cavity was opened, adhesions between the visceral organs and abdominal wall were dissected, and the hernial sac was excised. The length and width of the fascial defect were measured in cm intraoperatively and recorded. The posterior rectus sheath was separated from the rectus muscle to create the space for the mesh, taking care to prevent injuries to the epigastric vessels and neurovascular bundles. Transversus abdominis release (TAR) was performed when the previous dissection alone was not enough to guarantee adequate closure of the midline. The posterior rectus sheath was closed without tension with a small-bite technique and slowly absorbable 2/0 monofilament running sutures. The rule of a suture-to-wound length ratio of at least 4:1 was respected [11]; consequently, the total number of running sutures depended on the length of the defect. The mesh (ProGrip™, Medtronic) was placed below the rectus muscle with the self-gripping surface down and with an overlap of at least 5 cm. In two cases, two meshes were used to cover the entire defect by overlapping them. A drainage tube was placed in the retromuscular space above the mesh. The anterior rectus sheath was closed with the same technique used for the posterior sheath. The subcutaneous tissues were sutured with absorbable stitches, and the skin was stapled. When there was a large subcutaneous dead space, a drain was placed. The drains were removed when the daily drainage volume was less than 40 ml. Patients were followed in the outpatient clinic until they fully recovered, and early complications were recorded. Then, all patients were visited every 3 months, and when there was concern of recurrence, a CT scan was performed.

### Statistical analysis

Descriptive statistics were used to characterize the study population. Continuous data were analysed as the means (with SD and range) and median (with range). Categorical data were analysed as frequencies and percentages.

## Results

This retrospective analysis enrolled all 37 patients (17 females and 20 males) with IHs who were treated with this technique at our department from June 2016 to November 2018. Their mean age and mean body mass index were 58 ± 13 years (median 57 years; range 13–85 years) and 27 ± 12.5 kg/m^2^ (median 26.3 kg/m^2^; range 18–35 kg/m^2^), respectively. The demographic data are reported in Table 1. There were nine cases of IH recurrence. According to the EHS classification, the IHs were classified as M2M3M4 in 13 patients (35.2%) and as W2 in 19 patients (51.4%) (Table 2). The size of the mesh was 15 × 15 cm^2^ in 14 patients, 20 × 15 cm^2^ in 12 patients, and 30 × 15 cm^2^ in 9 patients; in 2 patients, 2 meshes were used to cover the entire defect. TAR was performed in seven patients (18.9%). The mean hospital stay was 5.9 ± 2.1 days (median 5 days; range 3–12 days). The postoperative complications are shown in Table 3. Seroma occurred in two patients (5.4%) and was treated with fluid aspiration; wound dehiscence (two patients) and haematoma (two patients) also occurred and were treated conservatively. The patients’ follow-up compliance was 100%. After a mean follow-up duration of 18.1 ± 6.7 months (median 16 months; range 10–39 months), two patients (5.4%) suffered from occasional mild pain, and there were three cases (8.1%) of recurrence. One was due to mesh damage in the central part, which was discovered during subsequent repair due to recurrence. Another patient refused the operation because he was asymptomatic, and the third patient had an abdominal *aortic aneurysm*, so IH repair was postponed. No movement limitations, SSIs or mesh infections were reported by any of the patients (Table 3).

**Table 3:** Postoperative outcomes

Early postoperative complications (total)	6 (16.2%)
Seroma	2 (5.4%)
Haematoma	2 (5.4%)
Wound dehiscence	2 (5.4%)
Mean hospital stay (days)	5.9 ± 2.1 days (median 5 days; range 3–12 days)
Mean follow-up (months)	18.1 ± 6.7 months (median 16 months; range 10–39 months)
Late postoperative complications (total)	5 (13.5%)
Recurrence rate	3 (8.1%)
Occasional mild pain	2 (5.4%)
Movement limitations	0 (0%)

### A systematic review of the literature

#### Materials and methods

**Fig. 1:**
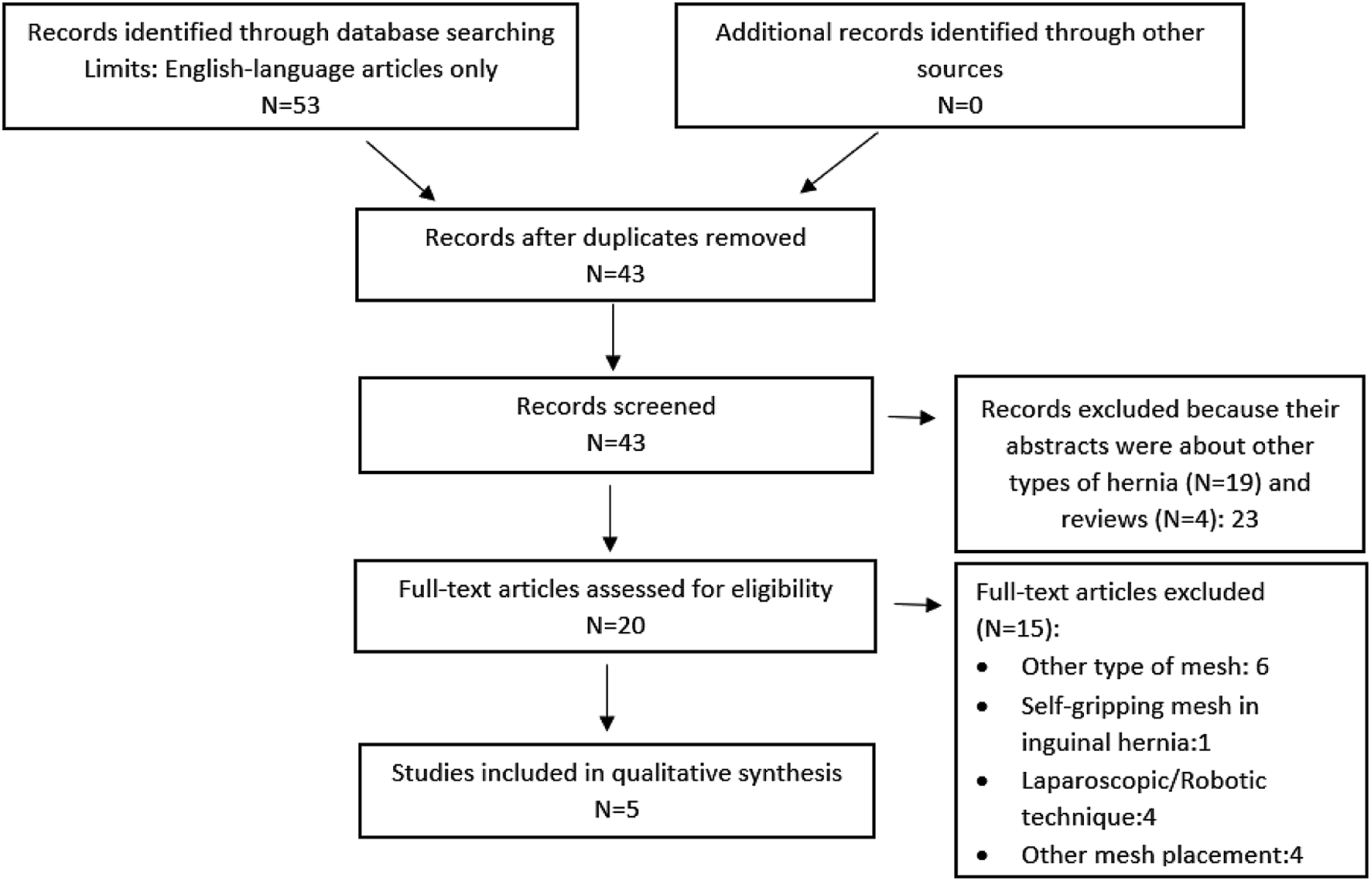
Prisma flow diagram of the study selection

A systematic review of the available English articles was conducted by two authors in January 2020 using PubMed, PubMed Central, Scopus and the Cochrane Library. The work was reported in line with PRISMA (Preferred Reporting Items for Systematic Reviews and Meta-Analyses) and AMSTAR (Assessing the methodological quality of systematic reviews) guidelines. The following search terms were used: “self-gripping”, “mesh”, “ventral hernia” or “incisional hernia”. All types of studies were included. The abstracts of 43 articles were screened (Fig. 1). For this analysis, five publications were considered relevant (Table 4).

**Table 4:** Results of incisional hernia (IH) repair with self-gripping mesh using the sublay technique

References	Type of study	Patients	Inclusion criteria	Defect size	Mesh placement	Postoperative complications	Hospital stay (days)	Recurrence	Follow-up
Khansa [12]	Retrospective	14	IH, elective setting	7.5 cm	Sublay	0	5.6	0	674 days
Bueno-Lledo [13]	Prospective	25	IH, elective setting	86 ± 28 cm^2^	Sublay	11.1%	5.8	0	13 months
Kroese [16]	Retrospective	46	IH, elective setting	0–4.99 cm (26%)	Sublay	22%	5	5.1%	25 months
				5–9.99 cm (35%)					
				> 10 cm (37%)					
				Unknown (2.2)					
Verhelst [14]	Retrospective	28	IH, elective setting	0–4.99 cm (29%)	Sublay	28.6%	5	0	12 weeks
				5–9.99 cm (21%)					
				> 10 cm (46%)					
				Unknown (3%)					
Harpain [15]	Retrospective	127 (111 IH)	IH, elective setting	9 cm	Sublay	28.3%	7	2.4%	11 months
Our experience	Retrospective	37	IH, elective setting	‹4 cm: 4 (10.8%)	Sublay	16.2%	5.9	8.1%	18 months
				≥ 4-10 cm:19(51.4%)					
				≥ 10 cm: 14 (37.8%)					

*NA* not applicable

### Results of the review

Four of the selected articles described the adopted surgical technique [12–15]. The extension of the dissection was not clearly reported, with the only exception being Bueno-Lledò et al. [13], who proposed a distance of 5–10 cm from the edges of the defect. The anterior and posterior sheaths were closed with continuous slowly absorbable sutures [12–15]. Microgrips were in contact with the posterior rectus sheath and not with the muscle [12–16], and drains were usually placed in the retrorectus space or were placed subcutaneously.

Khansa et al. [12] compared the outcomes of IH repair with sutureless and self-gripping mesh to those of transfascial fixation sutures with synthetic or biological mesh. Differences in the surgical site occurrence (SSO), length of hospital stay and recurrence rate between the two techniques were not statistically significant. However, patients treated with self-adhering mesh needed lower quantities of narcotics than other patients during hospitalization. Verhelst et al. [14] reported the absence of pain in 82% of patients and only moderate and mild pain in 11% and 7% of patients, respectively, with no reports of severe pain. Additionally, Bueno-Lledò et al. [13] demonstrated that self-adhering mesh allowed better outcomes than polypropylene mesh fixed with sutures. In fact, in this study, both postoperative pain and the rate of postoperative complications (haematoma) were significantly lower in patients treated with self-gripping mesh than in those treated with other types of prostheses. Kroese et al. [16] conducted a study in 46 patients with IHs treated with self-gripping mesh and reported that this technique was a safe procedure, with rates and mesh-related complications of 5.1% and 7.7%, respectively, after a mean follow-up period of 25 months. Conversely, Harpain et al. [15] observed a higher rate of postoperative complications in the self-gripping mesh group (28.3%) than in the non-self-gripping mesh group (13.7%). This difference was particularly evident when surgical site infection (SSI) and SSO were assessed. Therefore, 17.3% of patients developed seroma in the self-gripping mesh group versus 6.8% of patients in the other group. Similarly, the incidence of haematoma was significantly higher in the self-gripping mesh group than in the other group. Furthermore, there was no difference in terms of the length of hospital stay or the recurrence rate between the two groups.

Regarding the operative time, two studies [13, 15] demonstrated a slight reduction when self-gripping mesh was used instead of other types of mesh, but this difference was not statistically significant.

## Discussion

IH is the most frequent complication after laparotomy [17]. Sublay mesh placement seems to be the best option because it allows tension-free closure [13] and allows the prosthesis to be pushed towards the muscles by abdominal pressure [18]. In addition, the dissection creates a large space inside the abdominal wall where the mesh can be placed with adequate overlap, and the mesh is located in a highly vascularized area, facilitating the process of mesh integration. Moreover, this position is particularly advantageous because it reduces the risk of bowel adhesions [13, 17].

In recent years, the introduction of a novel self-gripping mesh has led to some changes in surgical techniques. In fact, because of the absorbable microgrips that adhere to tissues, this mesh does not require fixation sutures [8], thus avoiding the presence of gaps among them [19], which seems to be connected to an increased risk of recurrence [19]. Moreover, the absence of fixation points makes mesh placement easy and quick. According to Khansa et al. [12], the surface of contact between the mesh and the fascia is more extensive and consistent when there are no sutures that tend to create folds. This property leads not only to a reduction in the operative time but also to a decrease in postoperative pain compared to other types of mesh [13, 20]. In our opinion, the microgrips should be placed in contact with the rectus fascia for two main reasons. First, this avoids friction with the muscles, which could result in bleeding or pain; in fact, only two of our patients reported occasional mild pain. Second, the sheath offers a flatter plane than the muscles, allowing absolute adhesion of the entire mesh surface to the sheath and avoiding the formation of folds during the approximation of the rectus to the midline. Moreover, this type of mesh should be used only when the posterior sheath can be completely closed without tension to avoid contact between the microgrips and the abdominal organs, which could lead to complications.

Overall, the mean hospital stay length was 5.9 days in our experience, which was in line with that reported in the literature (Table 4). No statistically significant differences have been described between the duration of hospital stay after IH repair with ProGrip and other meshes fixed transfacially [12, 13, 15].

Regarding the postoperative complications (16.2%) reported in our study, the low rate of seroma (5.4%) was probably due to the use of suction drains, which prevented the formation of fluid collection and the consequent infection. Additionally, the sublay position seems to reduce the risk of infections derived from the skin, as infections were absent in our patients. During the median follow-up period of 16 months, there was a recurrence rate of 8.1% (3 patients), which is similar to or lower than that reported in the literature regarding the sublay technique [21]. One of the reasons for the low rate of recurrence could be that a large overlap ensures an adequate surface for tissue ingrowth, which acts as a mounting point for abdominal muscles [22]. However, our recurrence rate was higher than that described in the five selected articles regarding retromuscular repair with self-gripping mesh. This difference could depend on two major factors. First, the defect size should be taken into account; in fact, our three patients with recurrence had a W3 preoperative defect, while Khansa et al. [12] and Bueno-Lledò et al. [13] considered only W2 hernias, and the mean hernia defect size was 9 cm in the patients reported by Harpain et al. [15]. Second, the follow-up duration should be considered; for example, Verhelst et al. [14] followed patients for only 12 weeks, with a recurrence rate of 0%, which was different from the 25 months of follow-up performed by Kroese et al. (recurrence rate 5.1%) [16].

## Conclusions

This is the first review published in the literature regarding the open sublay technique with self-gripping mesh. Undoubtedly, the experience of the surgical team is one factor that largely influences outcomes [22]. The major advantage of this technique is the easy and rapid placement of the mesh, which does not require fixation points. Even though one study [15] reported better outcomes after non-self-gripping mesh repair, especially regarding postoperative complications, the other studies and our reported experience demonstrate that the self-gripping mesh can be safely adopted in the sublay position for IH repair. However, additional studies are needed to further investigate the long-term effects.

## Data Availability

Available if requested.

## References

[1] Bosanquet DC, Ansell J, Abdelrahman T (2015). Systematic review and meta-regression of factors affecting midline incisional hernia rates: analysis of 14,618 Patients. PLoS ONE.

[2] Burger JW, Luijendijk RW, Hop WC (2004). Long-term follow-up of a randomized controlled trial of suture versus mesh repair of incisional hernia. Ann Surg.

[3] Kokotovic D, Gögenur I, Helgstrand F (2017). Substantial variation among hernia experts in the decision for treatment of patients with incisional hernia: a descriptive study on agreement. Hernia.

[4] Eriksen JR, Gögenur I, Rosenberg J (2007). Choice of mesh for laparoscopic ventral hernia repair. Hernia.

[5] Holihan JL, Nguyen DH, Nguyen MT (2016). Mesh location in open ventral hernia repair: a systematic review and network meta-analysis. World J Surg.

[6] Deerenberg EB, Timmermans L, Hogerzeil DP (2015). A systematic review of the surgical treatment of large incisional hernia. Hernia.

[7] Sevinç B, Okuş A, Ay S (2018). Randomized prospective comparison of long-term results of onlay and sublay mesh repair techniques for incisional hernia. Turk J Surg.

[8] https://www.medtronic.com/covidien/en-gb/products/hernia-repair/mesh-products.html#progrip-self-gripping-polyester-mesh.html

[9] Bresnahan E, Bates A, Wu A (2015). The use of self-gripping (Progrip™) mesh during laparoscopic total extraperitoneal (TEP) inguinal hernia repair: a prospective feasibility and longterm outcomes study. Surg Endosc.

[10] García Ureña MÁ, Hidalgo M, Feliu X (2011). Multicentric observational study of pain after the use of a self-gripping lightweight mesh. Hernia.

[11] Muysoms FE, Antoniou SA, Bury K (2015). European Hernia Society guidelines on the closure of abdominal wall incisions. Hernia.

[12] Khansa I, Janis JE (2016). Abdominal wall reconstruction using retrorectus self-adhering mesh: a novel approach. Plast Reconstr Surg Glob Open 23.

[13] Bueno-Lledó J, Torregrosa A, Arguelles B (2017). Progrip self-gripping mesh in Rives-Stoppa repair: are there any differences in outcomes versus a retromuscular polypropylene mesh fixed with sutures? A “case series” study. Int J Surg Case Rep.

[14] Verhelst J, de Goede B, Kleinrensink GJ (2015). Open incisional hernia repair with a self-gripping retromuscular Parietex mesh: a retrospective cohort study. Int J Surg.

[15] Harpain F, Wimmer K, Dawoud C, Ogrodny P, Stift A (2020). Short-term outcome after ventral hernia repair using self-gripping mesh in sublay technique—a retrospective cohort analysis. Int J Surg.

[16] Kroese LF, van Eeghem LHA, Verhelst J (2017). Long term results of open complex abdominal wall hernia repair with self-gripping mesh: a retrospective cohort study. Int J Surg.

[17] Pauli EM, Rosen MJ (2013). Open ventral hernia repair with component separation. Surg Clin N Am.

[18] Schumpelick V, Klinge U, Junge K (2004). Incisional abdominal hernia: the open mesh repair. Langenbecks Arch Surg.

[19] Hopson SB, Miller LE (2015). Open ventral hernia repair using ProGrip™ self-gripping mesh. Int J Surg.

[20] Suciu BA, Halmaciu I, Fodor D (2018). Comparative study on the efficiency of 2 different types of meshes (Polypropylene and ProGripTM ) in the surgical treatment of incisional hernias. Mater Plast.

[21] Köckerling F, Schug-Pass C, Scheuerlein H (2018). What is the current knowledge about sublay/retro-rectus repair of incisional hernias?. Front Surg.

[22] Kurzer M, Kark A, Selouk S (2008). Open mesh repair of incisional hernia using a sublay technique: long-term follow-up. World J Surg.

